# APOBEC3A Is Implicated in a Novel Class of G-to-A mRNA Editing in *WT1* Transcripts

**DOI:** 10.1371/journal.pone.0120089

**Published:** 2015-03-25

**Authors:** Ahmadreza Niavarani, Erin Currie, Yasmin Reyal, Fernando Anjos-Afonso, Stuart Horswell, Emmanuel Griessinger, Jose Luis Sardina, Dominique Bonnet

**Affiliations:** 1 Haematopoietic Stem Cell Laboratory, Cancer Research UK, London Research Institute, London, United Kingdom; 2 Digestive Disease Research Institute (DDRI), Shariati Hospital, Tehran University of Medical Sciences, Tehran, Iran; 3 Department of Haematology, University College London Hospitals NHS Trust, London, United Kingdom; 4 Department of Bioinformatics, Cancer Research UK, London Research Institute, London, United Kingdom; 5 INSERM U1065, Mediterranean Centre for Molecular Medicine (C3M), Université Nice Sophia Antipolis, Nice, France; 6 Instituto de Biología Funcional y Genómica, CSIC/Universidad de Salamanca, Salamanca, Spain; Cincinnati Children's Hospital Medical Center, UNITED STATES

## Abstract

Classic deamination mRNA changes, including cytidine to uridine (C-to-U) and adenosine to inosine (A-to-I), are important exceptions to the central dogma and lead to significant alterations in gene transcripts and products. Although there are a few reports of non-classic mRNA alterations, as yet there is no molecular explanation for these alternative changes. *Wilms Tumor 1 (WT1)* mutations and variants are implicated in several diseases, including Wilms tumor and acute myeloid leukemia (AML). We observed two alternative G-to-A changes, namely c.1303G>A and c.1586G>A in cDNA clones and found them to be recurrent in a series of 21 umbilical cord blood mononuclear cell (CBMC) samples studied. Two less conserved U-to-C changes were also observed. These alternative changes were found to be significantly higher in non-progenitor as compared to progenitor CBMCs, while they were found to be absent in a series of AML samples studied, indicating they are targeted, cell type-specific mRNA editing modifications. Since *APOBEC/ADAR* family members are implicated in RNA/DNA editing, we screened them by RNA-interference (RNAi) for *WT1*-mRNA changes and observed near complete reversal of *WT1* c.1303G>A alteration upon *APOBEC3A (A3A)* knockdown. The role of A3A in mediating this change was confirmed by A3A overexpression in Fujioka cells, which led to a significant increase in *WT1* c.1303G>A mRNA editing. Non-progenitor CBMCs showed correspondingly higher levels of *A3A*-mRNA and protein as compared to the progenitor ones. To our knowledge, this is the first report of mRNA modifying activity for an APOBEC3 protein and implicates A3A in a novel G-to-A form of editing. These findings open the way to further investigations into the mechanisms of other potential mRNA changes, which will help to redefine the RNA editing paradigm in both health and disease.

## Introduction

According to the central dogma, genetic information is faithfully transferred from DNA to mRNA to protein. Classic mRNA editing in both plant and animal cells is an important exception where mRNA is altered during or after transcription. In human cells, APOBEC1 (apolipoprotein B mRNA editing enzyme, catalytic polypeptide 1; A1) was first recognised to alter cytidine to uridine (C-to-U) in *APOB* (apolipoprotein B) transcripts, changing the Gln codon (CAA) to a nonsense codon (UAA), and leading to ApoB48 rather than ApoB100 variant[1]. ADAR1 (adenosine deaminase, RNA-specific) was then identified to modify adenosine in double-stranded (ds-) mRNA to inosine (A-to-I), causing unwinding of the ds-mRNA[2]. Since inosine shows similar base-pairing properties to guanosine (G), it is interpreted as G by the translation machinery as well as *in vitro* polymerase reactions. While *APOBEC1* knockout leads to defects which are not incompatible with development[3], ADAR1 is essential for maintenance of hematopoiesis[4], and its gene knockout leads to lethal hematopoietic impairment and liver disintegration in mouse embryos[5]. Other APOBEC family members are mainly known for their DNA-editing functions.

Both classic editing events in mammals comprising A-to-I and C-to-U alterations are mediated by nucleoside deamination reactions. Although there are some reports of reverse U-to-C[6, 7] as well as G-to-A[7, 8] alterations in mammalian transcripts, they cannot be explained by deamination reactions. Such non-classic changes are overlooked in computational studies or attributed to misalignment to the opposite strand[9], and many are systematically disregarded due to coincidence with polymorphic sites. A recent study reported different types of RNA-DNA differences (RDDs), including G-to-A and U-to-C changes in B lymphocytes[10], apparently making the editing paradigm much more complicated. However, due to the lack of a known molecular mechanism to effect these non-classic changes, the nature of such editing remains uncertain.

Non-classic U-to-C mRNA editing was first reported in *WT1* transcripts[6]. WT1 is a regulatory protein with dual tumor suppressor/oncogene activity depending on the isoforms expressed, including the Lys-Thr-Ser (KTS) variant. WT1 splicing variants with excluded tripeptide (-KTS) mainly act as transcriptional regulators, while the isoforms retaining the tripeptide (+KTS) show post-transcriptional activity (reviewed in [11]). In addition, *WT1* mutations affecting the zinc finger (ZnF) domains are implicated in Wilms tumor[12] and acute myeloid leukemia (AML)[13]. While studying the role of *WT1* variants in AML and CBMCs, we observed recurrent G-to-A and occasional T-to-C changes in *WT1*-cDNA from CBMCs. We first demonstrated that such alternative changes were true cell type-specific mRNA modifications, rather than *in vitro* phenomena. Next, we hypothesized that known RNA/DNA editors might be implicated in these modifications. Hence, we used these novel changes in *WT1* as a marker and assessed how they were affected by *APOBEC/ADAR* knockdown. The results were confirmed by overexpression studies.

## Materials and Methods

### CBMC collection

Human umbilical cord blood samples were collected from normal deliveries in the Royal London Hospital after obtaining written and signed informed consent, and with the project approved by the East London Research Ethics Committee. The samples were processed separately (Cb samples), or up to five samples were pooled for some experiments (Cbp samples). Blood was diluted using 2 volumes of phosphate buffered saline (PBS) before being layered on half volume of Ficoll in 50 ml tubes and centrifuged at 360 X *g* for 30 minutes at 20°C. The middle buffy coat layer containing the CBMCs was transferred to a new tube and washed with PBS containing 2% fetal bovine serum (FBS), before spinning at 300 X *g* for 7 minutes at 4°C. Red cell lysis was performed using cold ammonium chloride. Viable cells were counted by Trypan blue exclusion on a Neubauer hemocytometer.

### Cell separation

Progenitor (lineage-marker negative) CBMCs were separated from non-progenitor (lineage-marker positive) cells using the StemSep Human Progenitor Enrichment Kit (StemCell Technologies) according to the manufacturer’s protocol. Briefly, CBMCs were incubated with a cocktail of antibodies against human hematopoietic lineage markers, followed by incubation with a magnetic colloid. The cell suspension was then pumped through a negative selection column mounted in a magnetic stand. The progenitor CBMCs were collected at the tubing outlet, and the non-progenitor CBMCs were separately washed through the column after removal from the magnetic stand. Cells were cryopreserved in 10% DMSO / 90% FBS at -80°C for later use.

### AML samples

Peripheral blood or bone marrow samples were collected from AML patients attending St Bartholomew’s Hospital, London, after obtaining written and signed informed consent. Established myeloid cell lines THP1[14], Fujioka[15], HL60[16], and KG1[17] were obtained from London Research Institute’s repository.

### DNA and RNA extraction

Genomic DNA was extracted from 10^6^ cells using DNeasy Blood & Tissue Kit (Qiagen) according to manufacturer’s protocol. Total RNA was also extracted from up to 5 x 10^5^ and 5–50 x 10^5^ cells using RNeasy Micro and Mini Kits (Qiagen), respectively, according to manufacturer’s protocols. All RNA samples were treated with DNase, as recommended by the manufacturer.

### PCR

*WT1* exon 7 was amplified using 20–50 ng of gDNA, 200 nM Ws-X7f5 (see S3 Table for primers), 200 nM Ws-X7r5, 200 μM dNTP mix, and 1 U Taq DNA Polymerase (Qiagen) on a PTC-225 DNA Engine (MJ Research) for 35 cycles with annealing at 61°C. *WT1* exon 9 was also amplified according to the same protocol, but using Ws-X9f3 and Ws-X9r2 primers. The amplicons were then gel-extracted using Qiaquick Gel Extraction Kit (Qiagen), making it ready for cloning or direct Sanger sequencing.

### RT-PCR

cDNA was made according to the SuperScript III First-Strand Synthesis protocol (Life Technologies) using up to 5 μg total RNA, 2.5 μM oligo(dT)_20_, and 500 μM dNTP mix. *WT1*-cDNA was amplified using a mixture of 2 μl cDNA, 300 nM Ws-Ex6f (see S3 Table for primers), 300 nM Ws-Ex10r, 200 μM dNTP mix, and 1 U Taq DNA polymerase (Qiagen) on PTC-225 DNA Engine (MJ Research) for 35 cycles with denaturation and annealing temperatures of 93°C and 58°C, respectively. The amplicons were then gel-purified as described above.

### Quantitative RT-PCR

*A3A* and *GAPDH* (glyceraldehyde-3-phosphate dehydrogenase) mRNA levels were assessed using Taqman Gene Expression assays (Hs00377444_m1 and Hs99999905_m1, respectively; Life Technologies) according to manufacturer’s protocol. *A3A* and *GAPDH* mRNA levels were assessed in triplicate. The Life Technologies 7900HT Fast Real-Time PCR System was used for amplification, and the results were analyzed using SDS software (v2.3, Life Technologies).

### gDNA and cDNA Cloning

Gel purified PCR or RT-PCR products were cloned using TOPO XL PCR Cloning Kit (Life Technologies) according to manufacturer’s instructions. Transformed *E*. *coli* was spread on LB agar plates at 37°C overnight. Between 30 to 50 colonies per RT-PCR product or 280 colonies per PCR product were randomly picked and inoculated into 1 ml LB media in 96-well blocks, and incubated in a shaker at 37°C overnight for plasmid preparation. In order to assess other hematopoietic transcripts in CBMCs and also *WT1-*cDNA in AML cells, at least 10 colonies were examined per sample. The plasmids were extracted using Nucleospin Robot-96 Plasmid Core Kit (ClonTech) on a BioRobot 9600 machine (Qiagen).

### Sequencing

Direct Sanger sequencing was performed using gel-purified PCR/ RT-PCR products, the same amplification primers, and BigDye Terminator kit (v3.1, Life Technologies), for 25 cycles. The amplified gDNA/ cDNA was analyzed using a 3730xl BioAnalyzer (Life Technologies) after dye-exclusion with Chemagic SEQ Pure Kit on a Biomek FX machine (Beckman Coulter). Sanger sequencing of the gDNA/ cDNA clones was performed using the same protocol, but with miniprep plasmids and universal M13f/ M13r primers for the cycling reaction.

### siRNA design

One siRNA (small-interfering RNA) was designed for each of 14 candidate genes using Dharmacon’s siDesign Center (www.dharmacon.com/designcenter) and two additional siRNAs for *A3A*. The siRNAs were selected using the following criteria: a GC ratio of 30–64%; preferential targeting of the open reading frame (ORF); affecting as many variants as possible; highest predicted efficiency; at least two mismatches for each of the antisense and sense strands; and low frequency of seed region (sense nucleotides 2 to 8) matching to 3’UTRs throughout the genome (S2 Table). Two control siRNAs were designed with no specific target in human transcriptome, of which one was linked to Alexa-488 fluorochrome for microscopic assessment of the transfection efficiency.

### siRNA transfection

Pooled or separate CBMC samples were resuspended at a density of 5 x 10^5^ cells /ml in 5% FBS-IMDM (Iscove's Modified Dulbecco's Medium, Life Technologies) and incubated at 37°C and 5% CO_2_ overnight. For silencing experiments, a mixture of 3 μl test or control siRNA 20 μM (Sigma-Aldrich; final concentration 100 nM), 3 μl Alx-si 20 μM (Sigma-Aldrich), and 5 μl HiPerfect Transfection Reagent (Qiagen) was added to 8 x 10^5^ CBMCs in 109 μl 5% FBS-IMDM in a 24-well plate and incubated at 37°C and 5% CO_2_ for 6 hours. Then, they were topped up using 480 μl pre-warmed 5% FBS-IMDM and incubated as above. The plates were examined using fluorescent microscopy for transfection efficiency after 24 hours. Efficiently transfected samples were harvested 48 hours after the transfection, and their total RNAs extracted immediately as described above.

### Plasmid transfection

Green-fluorescent protein (GFP)-tagged *A3A* overexpression (RG220995) and empty pCMV6-AC-GFP (PS100010) vectors were obtained from OriGene. Overexpression was performed using the Amaxa Cell Line Nucleofector Kit V (Lonza) with 3.5ug DNA per 2 million cells. GFP positive cells were sorted on a Cytomation MoFlo or a BD FACS Aria sorter.

### Western blot analysis and protein quantitation

Protein was extracted using RIPA buffer containing protease inhibitor and DTT (Sigma-Aldrich). Western blots were performed using a Novex Mini Cell gel tank and Novex gels (Life Technologies), and semi-dry transfers using Hybond ECL membrane (GE Healthcare). Protein was detected using rabbit anti-APOBEC3A (Santa Cruz Biotechnology) and mouse anti-beta-Actin (Sigma-Aldrich), followed by HRP-conjugated goat anti-rabbit and anti-mouse secondary antibodies (Sigma-Aldrich), respectively. Detection was performed using ECL Plus (GE Healthcare). Relative protein quantification was performed using Image J.

### Statistical analysis

A% value at c.1303 was obtained by counting the A/G in sequenced clones and performing the calculation 100 x A / (A + G). Fisher’s Exact test (GraphPad Prism, version 5.01) was used to compare the A% at c.1303 between each experiment and the control, and also for alterations in progenitor/ non-progenitor subpopulations. For association analyses, under a hypothesis of independence of changes occurring at the two sites, the count data for changes at neither, only at site 1, only at site 2, and at both sites follow a multinomial distribution. We estimated the independent probabilities from the observed data (i.e. total number of changes at each site relative to total number of clones assessed) and provided the R function dmultinom with these estimates to assess the probability of the observed data under this null.

## Results

### *WT1* transcripts are subject to alternative changes

**Fig 1:**
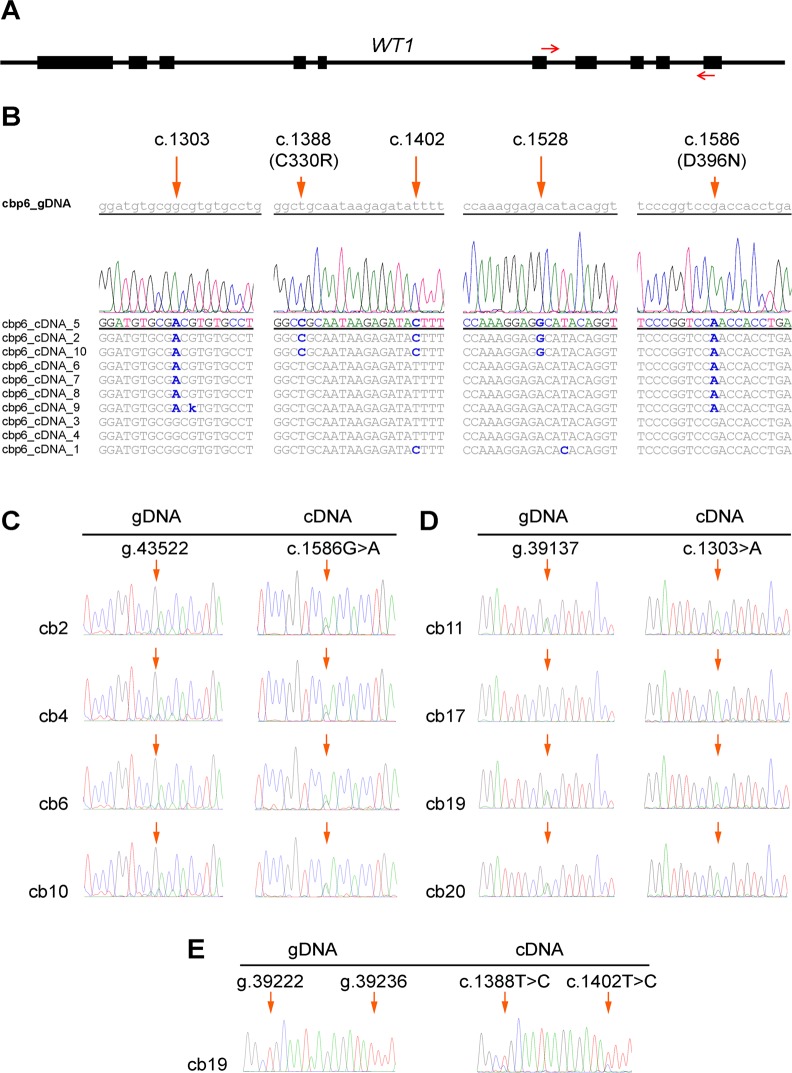
Identification and validation of alternative *WT1* mRNA changes. (A) *WT1* gene and the 400 bp cDNA amplicon between exons 6 and 10, as indicated by arrows. (B) *WT1*-cDNA clonal changes in a pool of CBMC cells are shown, with one aligned chromatogram expanded and paired gDNA sequence. In addition to one classic A-to-G change at c.1528, two alternative G-to-A alterations are seen at c.1303 and c.1586, and two alternative T-to-C changes can also be seen at c.1388 and c.1402. (C) Major G-to-A changes at c.1586 in four samples, with no A allele in paired gDNA. (D) Major G-to-A alterations at c.1303 (common A/G SNP site) in three heterozygous CBMC samples (cb11, cb19, and cb20), and nearly complete G-to-A change in one homozygous GG one (cb17). (E) Partial T-to-C (U-to-C in mRNA) changes at c.1388 and c.1402 in one sample. Altered sites are indicated by arrows in both gDNA and cDNA chromatograms.

Exons 6 to 10 of *WT1-*cDNA (Fig. 1A), corresponding to ZnF1 to ZnF4 domains, were cloned and sequenced from a pooled sample of human CBMCs, representative of hematopoietic cells, to investigate potential mRNA variants affecting WT1 function. Differences were observed at single-nucleotide cDNA sites, as compared to the EST (Expressed Sequence Tag) and RefSeq databases, and paired DNA sequence (Fig. 1B). These differences included a classic A-to-G change at c.1528 (NM_024426.3), novel T-to-C alterations at c.1388 and c.1402, and also novel G-to-A changes at c.1303 and c.1586, among them two modifying the amino acid sequence of the WT1 (c.1388T>C and c.1586G>A resulting in p.C330R and p.D396N, respectively). The c.1303 position corresponds to a common A/G polymorphic site (rs16754; g.39137, NG_009272.1), but there was no genomic A allele in the sample. We refer to these potential G-to-A and U-to-C mRNA alterations as alternative mRNA changes, to distinguish them from classic deamination ones.

BLAST analysis of the *WT1* amplicon showed little homology to any other known ESTs, including antisense ESTs and *WT1* antisense (NCBI Gene ID: 51352). This excluded the possibility of the classic C-to-U and A-to-G alterations in an antisense strand as a cause of observed G-to-A and U-to-C changes, respectively. We tested the possibility of minor primary genomic changes samples with prominent c.1586G>A alteration, which did not show any genomic DNA (gDNA) changes in 280 copies. In order to validate the alternative modifications in a larger series of CBMCs, we later examined the same *WT1* region using Sanger sequencing of paired gDNA-cDNA from a series of 21 CBMC samples (S1 Table). Sixteen samples showed prominent G-to-A change at c.1586 (Fig. 1C). Among nine samples with an informative AG genotype at rs16754 (corresponding to c.1303), eight showed an exclusive or predominant A in the cDNA (Fig. 1D; cb11). All nine samples with AA genotype showed just A at cDNA level, and more importantly, prominent A was found in the cDNA of three homozygous GG samples (Fig. 1D; cb17), which excluded the allele-specific gene expression as a potential implicated mechanism. Overall, both c.1303 and c.1586 target sites showed variable levels of G-to-A change in about two thirds of the informative samples studied (Fig. 1C, D). Moreover, two CBMC samples showed additional alternative c.1388T>C and c.1402T>C changes (Fig. 1E). These alternative mRNA changes therefore appear to be recurrent in CBMCs.

### *WT1* mRNA alternative changes show a cell type-specific pattern

**Fig 2:**
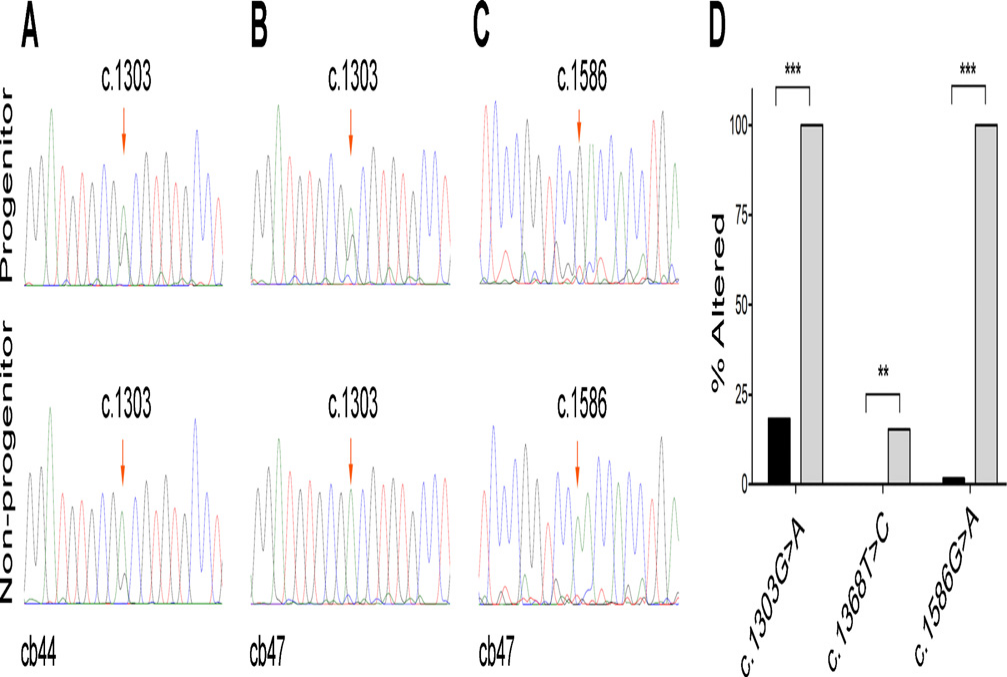
Differential mRNA changes in CBMC progenitor and non-progenitor subpopulations. (A) Sanger sequencing of *WT1*-cDNA in subpopulations of cb44 shows that G-to-A alteration at c.1303 is grossly higher in non-progenitor as compared to progenitor subpopulation. G-to-A alteration at both c.1303 (B) and c.1586 (C) were also found to be higher in the non-progenitor subpopulation of cb47 sample, as compared to the progenitor one. (D) Sequencing of the *WT1*-cDNA clones in cb47 subpopulations confirms the differential changes at c.1303 and c.1586, and also shows a minor T-to-C change at c.1368 which is significantly higher in non-progenitor as compared to the progenitor subpopulation. ** *P* < 0.01. *** *P* < 0.001.

To test the variability of these changes among different cell types, we examined the cDNA from eight CBMC samples sorted into progenitor and non-progenitor subpopulations. These samples showed grossly increased alternative changes in non-progenitor compared to progenitor CBMCs at c.1303G or c.1586G (Fig. 2A-C), which was subsequently confirmed by Sanger sequencing of the cDNA clones (Fig. 2D). Furthermore, an assessment of a series of 19 leukemic samples by cloning did not show any repeating G-to-A mRNA changes at c.1586 (S1 Fig.). There was also no repeating G-to-A change at c.1303 in those samples with informative heterozygous genotypes. The fact that observerd changes repeated at high levels at particular sites and in a sample-specific manner (RNA vs. DNA, CBMC vs. leukemic cell, non-progenitor vs. progenitor cell) essentially excluded the implication of technical errors inherent to Taq polymerase and reverse transciptase enzymes in PCR, RT-PCR, and sequencing reactions. The presence of separate clones in clonal sequencing further excluded the sequencing error as a cause of the observed alterations.

### A3A knock-down leads to nearly complete reversal of *WT1* c.1303G>A change

**Fig 3:**
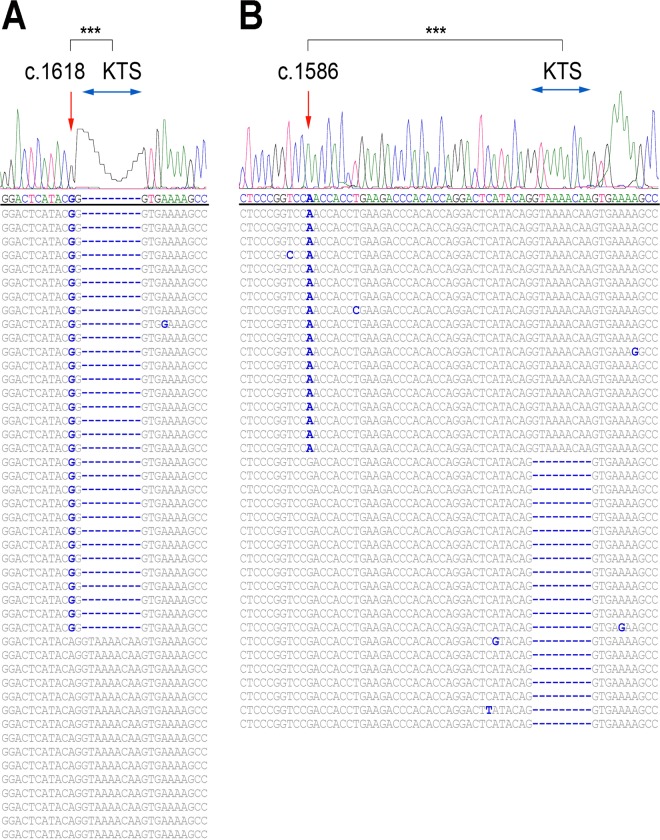
Association of mRNA changes with canonical *WT1* variants. (A) Sanger sequencing of the *WT1* cDNA clones shows that all classic c.1618A>G alterations are in *cis* with canonical-KTS isoforms. (B) All alternative c.1586G>A mRNA changes are in *cis* with functionally relevant +KTS variants. *** *P* < 0.001.

**Fig 4:**
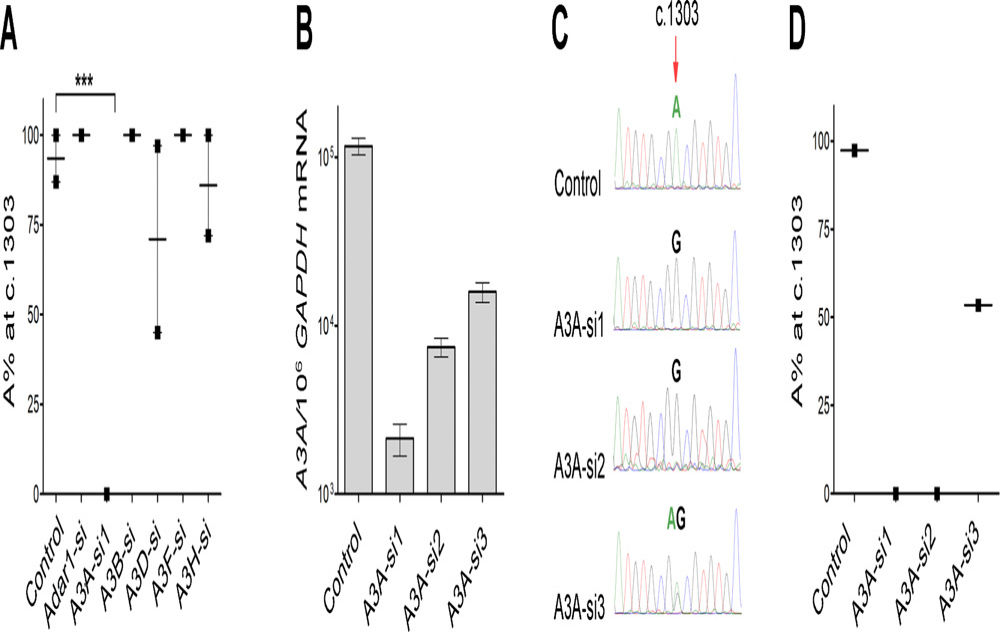
Screen of candidate genes by the ability of targeted siRNAs to reverse c.1303G>A alteration in CBMC samples. (A) Six selected genes were screened by specific siRNAs, and the resultant A% values at c.1303 were calculated from the *WT1* cDNA clones sequenced. A3A-si1 resulted in nearly complete reversal of c.1303G>A change, with no evidence of G-to-A alterations elsewhere. Values indicate the mean +/- SEM. (B) Silencing efficiencies of different *A3A*-siRNAs were assessed by Taqman gene expression assay in CBMCs. (C) Phenotypic analysis of corresponding *A3A-*silenced CBMCs using Sanger sequencing of the *WT1*-cDNA shows a virtually complete reversal of c.1303G>A change by A3A-si1 and A3A-si2, and a partial reversal by A3A-si3. (D) Quantification of the results in (C) above, as determined by Sanger sequencing of the *WT1*-cDNA clones, confirming qualitative data. *** *P* < 0.001.

Clonal analysis of *WT1-*mRNA alterations in CBMCs identified two novel classic (1618A>G) and alternative (1586G>A) changes to be significantly associated with canonical KTS splicing variants (Fig. 3), affecting protein sequence and function[18]. WT1 splicing variants retaining the KTS tripeptide bind preferentially to RNA and perform mRNA processing roles, while isoforms with excluded KTS bind preferentially to DNA and regulate transcription. Moreover, we observed a pattern of associated alternative-classic events. For example, c.1388T>C changes coincided (i.e. were in *cis*) with c.1528A>G ones (*P* = 0.0006; Fig. 1B) in one sample. We asked whether this close *cis* position suggested a role for classic mRNA/DNA editors in alternative editing. Therefore, we first screened all *APOBEC*/*ADAR* genes using one specific siRNA per gene (S2 Table). A pool of CBMCs was transfected using different siRNAs and *WT1*-cDNA clones were sequenced to assess G-to-A changes as a marker for RNAi effect. The G-to-A alteration was found to be conserved only at c.1303 in the negative control samples, though a low level G-to-A change (<10%) was also seen at c.1644 in two control samples. Moreover, some non-conserved G-to-A alterations were also seen in knockdown experiments, including those at c.1581 using A3G-si (13%) and at c.1586 using A3C-si (88%) (S2 Fig.). Hence, those siRNAs with less than 10% G-to-A alteration at c.1303 (Adar1-si, A3A-si1, and A3H-si) or other non-conserved sites (A3A-si1, A3B-si, A3D-si, and A3F-si) were selected for further experiments. In follow-up experiments testing these six candidate genes, A3A-si1 resulted in nearly complete reversal of the c.1303G>A change compared to the control (Fig. 4A), with no G-to-A alterations at non-conserved sites.

In order to confirm that the observed RNAi effects were *A3A*-specific, we knocked down the gene using two additional specific siRNAs, alongside A3A-si1. Assessment of *A3A* mRNA level in the *A3A*-silenced CBMC samples showed 86% to 98% silencing efficiency for the tested *A3A*-siRNAs (Fig. 4B). Phenotypic analysis of *WT1-*cDNA clones demonstrated a correlated reduction of c.1303G>A alteration as compared to the control (Fig. 4C, D). We also examined the A3A protein level and A% at c.1303 in four myeloid cell lines which were heterozygous at rs16754. This showed a low A% in those cell lines with a relatively low A3A level (THP1 and Fujioka) and a high A% in those with a relatively high A3A level (HL60 and KG1) (S2 Fig.).

### A3A over-expression is associated with significant increase in *WT1* c.1303G>A change

**Fig 5:**
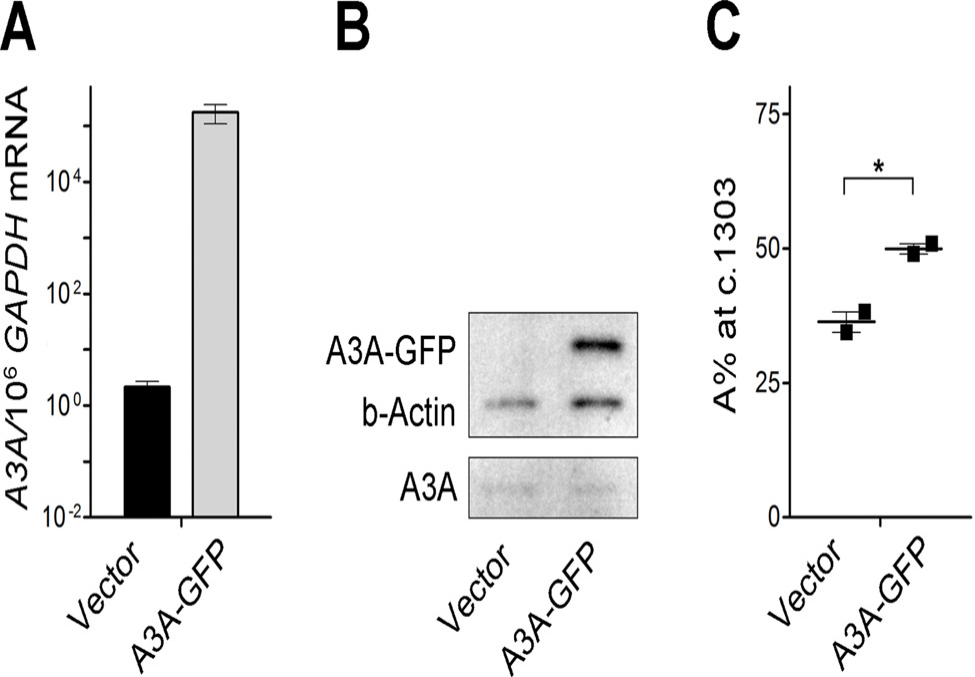
Validation of the role of A3A in c.1303G>A change using gene overexpression. (A) *A3A/*10^6^ *GAPDH* mRNA as assessed by Taqman gene expression assay in Fujioka/P31 cells 48 hours after transfection by A3A-GFP plasmid, as compared to the control vector. (B) Western Blot analysis of Fujioka cells transfected with control or A3A-GFP vector, showing exogenous GFP-tagged A3A, in addition to endogenous A3A. (C) Phenotypic analysis shows a modest effect of *A3A* overexpression in the induction of c.1303G>A change (i.e. increasing A% at c.1303) in Fujioka cells, as determined by Sanger sequencing of the *WT1*-cDNA clones. Values indicate the mean +/- SEM. * *P* < 0.05.

In order to exclude potential RNAi off-target effects, *A3A* was transiently over-expressed in the Fujioka cell line and the impact on mRNA editing examined. Basal *A3A* mRNA (Fig. 5A) and protein levels (Fig. 5B) increased significantly 48 hours after *A3A* transfection, which was associated with more than 1.5 fold increase in A% (from 36% to 54.5%; *P* = 0.015) at c.1303 (Fig. 5C).

### A3A expression is differentially higher in non-progenitor as compared to progenitor CBMCs

**Fig 6:**
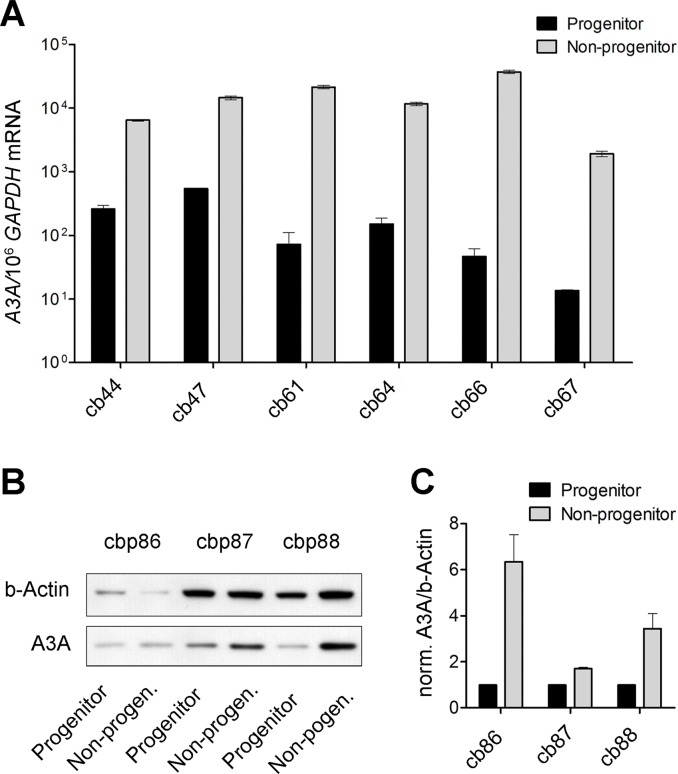
Differential expression of *A3A* mRNA in progenitor as compared to non-progenitor CBMCs. (A) Six CBMC samples were sorted into progenitor and non-progenitor cells, and *A3A* mRNA expression was assessed and normalized to *GAPDH* mRNA level. *A3A* expression in six pairs of samples was on average 228 (SD = 298) fold higher in non-progenitors as compared to progenitors, corresponding to differential G-to-A editing in these populations. (B) Three pairs of pooled CBMC samples were sorted into progenitor and non-progenitor subpopulations and examined for A3A level, showing higher levels in non-progenitors. (C) Quantitation of the A3A Western blot bands normalized to beta-Actin, confirming higher A3A levels in non-progenitor cells.

We finally tested whether the *A3A* levels correlated with the pattern of differential editing observed in CBMC populations. Assessment of *A3A* mRNA level among progenitor and non-progenitor cell populations showed a two-order higher level in the latter. Of note, the *A3A/GAPDH* mRNA ratio was between 2.0 x 10^-3^ and 36 x 10^-3^ in fresh non-progenitor CBMC subpopulations constituting more than 99% of the CBMCs, while it was just 1.4 x 10^-5^ to 54 x 10^-5^ in paired progenitor CBMC subpopulations (Fig. 6). A similar but less prominent difference was also seen in A3A protein level (Fig. 6B, C).

## Discussion

Non-classic G-to-A mRNA changes were first reported in *HNRNPK* (heterogeneous nuclear ribonucleoprotein K) transcripts in both malignant and normal colorectal samples[8], but later were also seen alongside non-classic U-to-C alterations in brain cell *TPH2* (tryptophan hydroxylase 2) transcripts[7]. G-to-A RNA editing has also been recently identified in the mushroom *Ganoderma lucidum* by RNA-sequencing[19]. Transamination and transglycosylation mechanisms have been proposed for plant U-to-C editing events in mitochondrial transcripts[20]. However, G-to-A change is probably more complicated, as G and A differ in two groups and no single reaction can interconvert them. Without a known mechanism, such changes are currently overlooked in computational studies or attributed to misalignment to the opposite strand[9], and many are systematically disregarded due to coincidence with polymorphic sites. A recent whole transcriptome study claimed to find widespread RNA-DNA differences (RDDs)[10], including G-to-A and U-to-C changes. However, no apparent correlations with ADAR1 expression levels were identified, and hence none of the “RDDs” could be attributed to a known regulated mRNA editing mechanism. Moreover, most “RDDs” were undermined later and attributed to misalignment of short sequencing reads to paralogous genomic sites[21] and systematic technical errors[22, 23].

Herein we report novel G-to-A mRNA changes in *WT1* transcripts at c.1303 and c.1586, which are of potential functional significance. The former corresponds to polymorphic site rs16754, which is a located in a hotspot for most *WT1* mutations in AML[13], and is also implicated in AML risk and *WT1* expression[24]. Moreover, germline and somatic mutations corresponding to c.1586G>A are implicated in Wilms tumor[12] and poor risk in AML[13], respectively, although it is reported to be polymorphic (rs28941778). We essentially excluded the possibility of antisense C-to-U changes mimicking sense G-to-A changes, and also minor DNA changes leading to prominent mRNA alterations. These alterations were found to be cell-type specific. For instance, they were differentially more prominent in nonprogenitor as compared to progenitor CBMCs. Although these cell type-specific differences indicate that alternative mRNA changes in CBMCs result from true biological alterations rather than an *in vitro* phenomenon, there is yet no known enzyme performing these novel changes.

Currently, APOBEC1 (A1), which classically deaminates cytidine to uridine (C-to-U), is the only known mRNA editor among APOBEC/AID family[1], with some DNA modifying activity when overexpressed[25]. Other family members including APOBEC3 proteins are mainly known for their single-stranded DNA (ssDNA) editing functions[26]. However, A3A has been recently reported to show even higher affinity to ssRNA than ssDNA[27]. Among the ADAR family, double-stranded mRNA editing activity is known for ADAR1[2] and ADAR2[28], which alter adenosine to inosine (A-to-I) with the inosine interpreted as guanosine.

Our clonal analysis of *WT1-*mRNA alterations revealed a pattern of associated alternative-classic events, including mutual *cis* G-to-A and C-to-U changes (S4 Fig.)(*P* = 2.6 x 10^-7^). Hence, we hypothesized that this *cis* position might suggest a role for classic mRNA/DNA editors in alternative editing. RNAi screening of the *APOBEC/ADAR* genes suggested A3A as the potentially implicated gene, and repeat experiments using two additional *A3A-*siRNAs led to similar results. These results were further tested by *A3A* overexpression in Fujioka cells, with relatively low A3A expression and informative AG genotype at c.1303, which led to a significant 1.5 fold increase in A% at that site. These findings confirm that the G-to-A class of alternative mRNA editing is specifically mediated by an *A3A*-dependent mechanism. However, the modest increase in G-to-A editing after *A3A* overexpression despite its almost complete reversal in RNAi studies may suggest a role for potential cofactors, as required by deaminases[29, 30].

This novel function raises a serious question that how a known deaminase, A3A, can be implicated in a non-deamination type G-to-A change. The fact that alternative changes were found to be associated with classic alterations, including mutual G-to-A and C-to-U changes (S4 Fig.), may suggest the involvement of a single common or two linked mechanisms for cytosine deamination and G-to-A change. On the other hand, the aminated form of G (2,6-diaminopurine) has been found to mimic A based on the base-pairing properties[31]. Hence, it can be assumed that the amine group resulted from C-to-U deamination or 5-methylcytidine deamination[32, 33] is shuttled to a linked reaction, altering G to 2,6-diaminopurine, mimicking A (S5 Fig.). However, the implication of base exchange (transglycosylation)[34] or nucleotide replacement can not be excluded in G-to-A alteration.

A3A has been found by previous studies to be highly expressed in monocytes/macrophages[35] and peripheral blood mononuclear cells[36]. Our results show that there is also a differential *A3A* expression in non-progenitor CBMCs (including monocytes/ macrophages) as compared to progenitors. This was in line with differential G-to-A editing activity observed in mentioned subpopulations (Fig. 2), further supporting the role of physiological levels of A3A in alternative mRNA editing.

While the human APOBEC3 (A3) family, consisting of seven proteins with one or two cytidinedeaminase (CDA) domains[37], have been characterized in many respects, this is the first report of an mRNA-editing function of A3A to our knowledge. Previously, A3 proteins have been shown to have selective antiviral activity against a range of viruses[38], constituting an innate barrier to viral nucleic acids[39]. More specifically, monodomain A3A passively enters the nucleus[40], restricts various ssDNA elements including 12retroviruses[41] and endogenous retro-elements *LINE-1* and *Alu*[40, 42], and removes foreign DNA[35]. Intriguingly, A3A was found to be an efficient 5-methylcytosine deaminase[43, 44], a process which is probably implicated in DNA demethylation[43]. Finally, ectopic *A3A* expression has been found to cause cell cycle arrest by inducing genomic hypermutation[32] and the DNA damage response (DDR)[45].

This novel function of A3A as a dual DNA-mRNA modifier raises broader questions of the role of mRNA editing, and specifically the role of A3A, in cancer. The fact that A3A induces particular changes in *WT1* mRNA which correspond to driver cancer mutations suggests a potential role for this novel modification in cancer mutagenesis. In fact, in a recent global study assessing 14 different cancer types, *A3B* and *A3A* expression was found to be significantly higher in those samples with an APOBEC mutation type, particularly in breast cancer samples[46]. Follow-up screening of the APOBEC family showed *A3B* and *A3A* expression levels correlated to the number of APOBEC mutations among different cancers studied[46). Moreover, another recent study found APOBEC mutational signature prevalent across 16 hematological and non-hematological malignancies[47]. Functional studies support this hypermutating effect of the A3B/A3A in breast cancer[48]. These studies suggest a significant role for A3A in cancer mutagenesis. However, whether this mutagenic activity is an off-target effect of a physiological role like viral restriction or alternative mRNA editing remains to be determined.

In summary, this study confirms the existence of two non-classic forms of mRNA editing in human, comprising G-to-A and U-to-C changes, using targeted Sanger sequencing rather than next-generation sequencing with its inherent potential for errors due to misalignment. These changes are likely to be of functional consequence in view of their cell specific nature, and in the case of G-to-A changes, their association with canonical *WT1* variants. More importantly, the G-to-A editing was specifically mediated by A3A, which to our knowledge, is the first report of a mechanism for alternative mRNA editing and furthermore of a function that has not previously been described for any of the APOBEC3 proteins. The implication of A3A in both mRNA and DNA alterations and its apparent up-regulation in different cancers raises the possibility that aberrations in alternative mRNA editing are involved in cancers. This is not only an intriguing question, but also a potential target for therapy. Moreover, our work demonstrates an approach to identify genes which mediate other potential novel RNA changes detected by modern high-throughput approaches. Finally, this study provides evidence against exclusion of the potential RNA editing sites which are simply coinciding with polymorphic sites in high-throughput sequencing studies.

## Supporting Information

S1 Fig Lack of *WT1* G-to-A changes at c.1586 in four representative AML samples. Nineteen AML samples were examined using Sanger sequencing of the *WT1*-cDNA clones, and the chromatograms were aligned and examined for potential changes at c.1586, which did not show any alterations.
(TIF)

S2 Fig Non-conserved G-to-A alterations associated with knockdown experiments. Knockdown experiments of the candidate genes were associated with some G-to-A alterations at non-conserved sites which were not observed in the control experiments, including changes at c.1581 (A) and c.1586 (B) associated with A3G-si and A3C-si, respectively.
(TIF)

S3 Fig Correlation of A% at c.1303 with A3A protein level in myeloid cell lines. A. A% of at c.1303 in four myeloid cell lines as determined by clonal sequencing of the *WT1* transcripts. B. Expression levels of A3A in corresponding myeloid cell lines compared to b-Actin as determined by Western blotting.
(TIF)

S4 Fig *WT1*-cDNA changes in CBMCs showing a pattern of associated alternative-classic events. Sanger sequencing chromatograms of the *WT1-*cDNA clones are aligned and examined for the changes occurring in the same clones, i.e. in *cis*, with one altered chromatogram expanded for each of the associated events. Statistically significant associations are found for c.1388U>C and c.1528A>G (A), as well as c.1505C>U and c.1609G>A (B and C). *** *P* < 0.001.
(TIF)

S5 Fig Models for classic and alternative mRNA changes. Classic mRNA editing is explained by deamination reactions converting C to U (A) and A to I, which mimics G (B), while proposed alternative mRNA editing model involves amination reactions converting U to C (C) and G to D, mimicking A (D). Each edited base is shown with its paired base.
(TIF)

S1 Table Summary of G-to-A mRNA changes in 21 CBMC samples at c.1303 and c.1586 positions, as identified by Sanger sequencing of paired DNA-cDNAs. Gross G-to-A changes were seen in seven informative samples at c.1303 (underlined), including three homozygous GG samples (cb5, cb17, and cb21) with prominent A at c.1303, not explained by imbalanced allele expression. Sixteen samples showed prominent A at c.1586 (underlined). Overall, 17 samples showed G-to-A change at c.1303 and/or c.1586. * Samples with additional T-to-C alterations at c.1388 and c.1402.
(PDF)

S2 Table Characteristics of the siRNAs used for screening of the implicated gene in alternative G-to-A mRNA editing. (PDF)

S3 Table Characteristics of the primers used for amplification of *WT1-*cDNA and DNA. (PDF)
