# Supplementary figures and images for: APOBEC3A Is Implicated in a Novel Class of G-to-A mRNA Editing in WT1 Transcripts
Source: PLoS One. 2015 Mar 25;10(3):e0120089. doi: 10.1371/journal.pone.0120089 (PMC4373805; doi:10.1371/journal.pone.0120089)

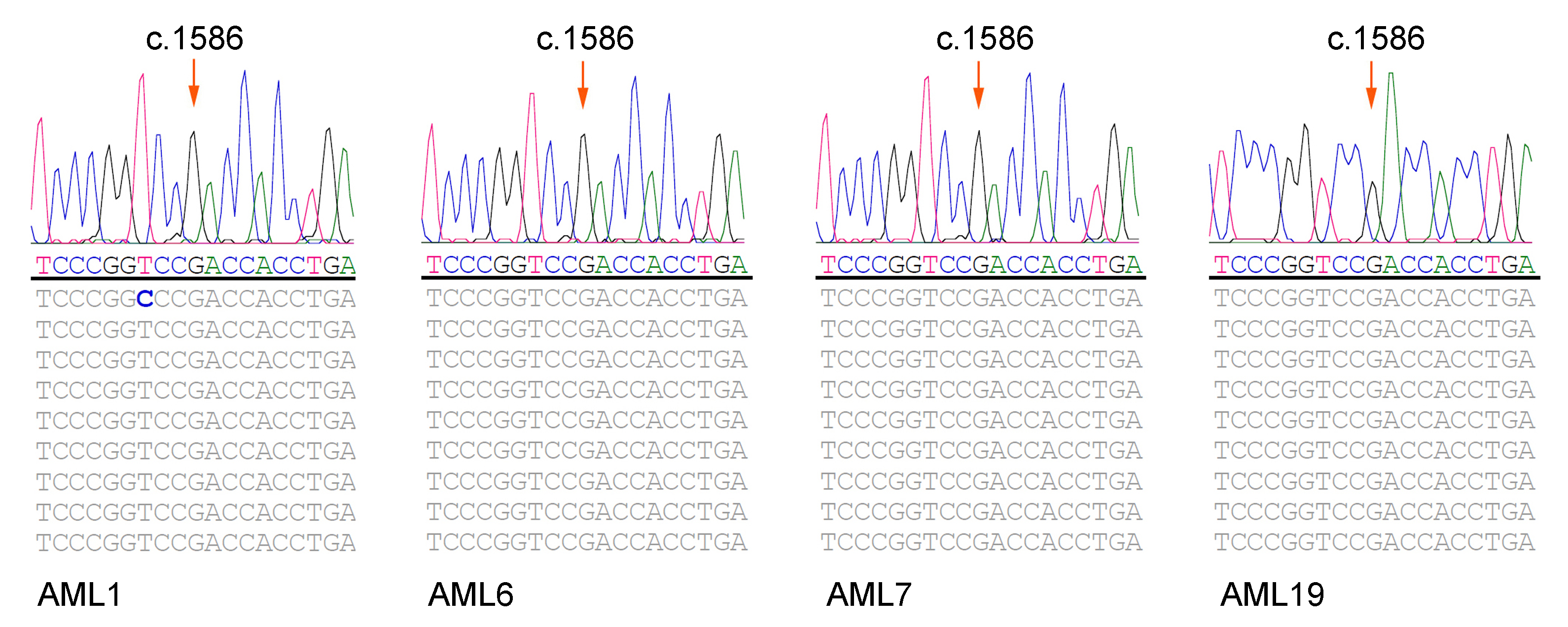

Supplement: S1 Fig — Nineteen AML samples were examined using Sanger sequencing of the WT1-cDNA clones, and the chromatograms were aligned and examined for potential changes at c.1586, which did not show any alterations. (TIF) [file pone.0120089.s001.tif]

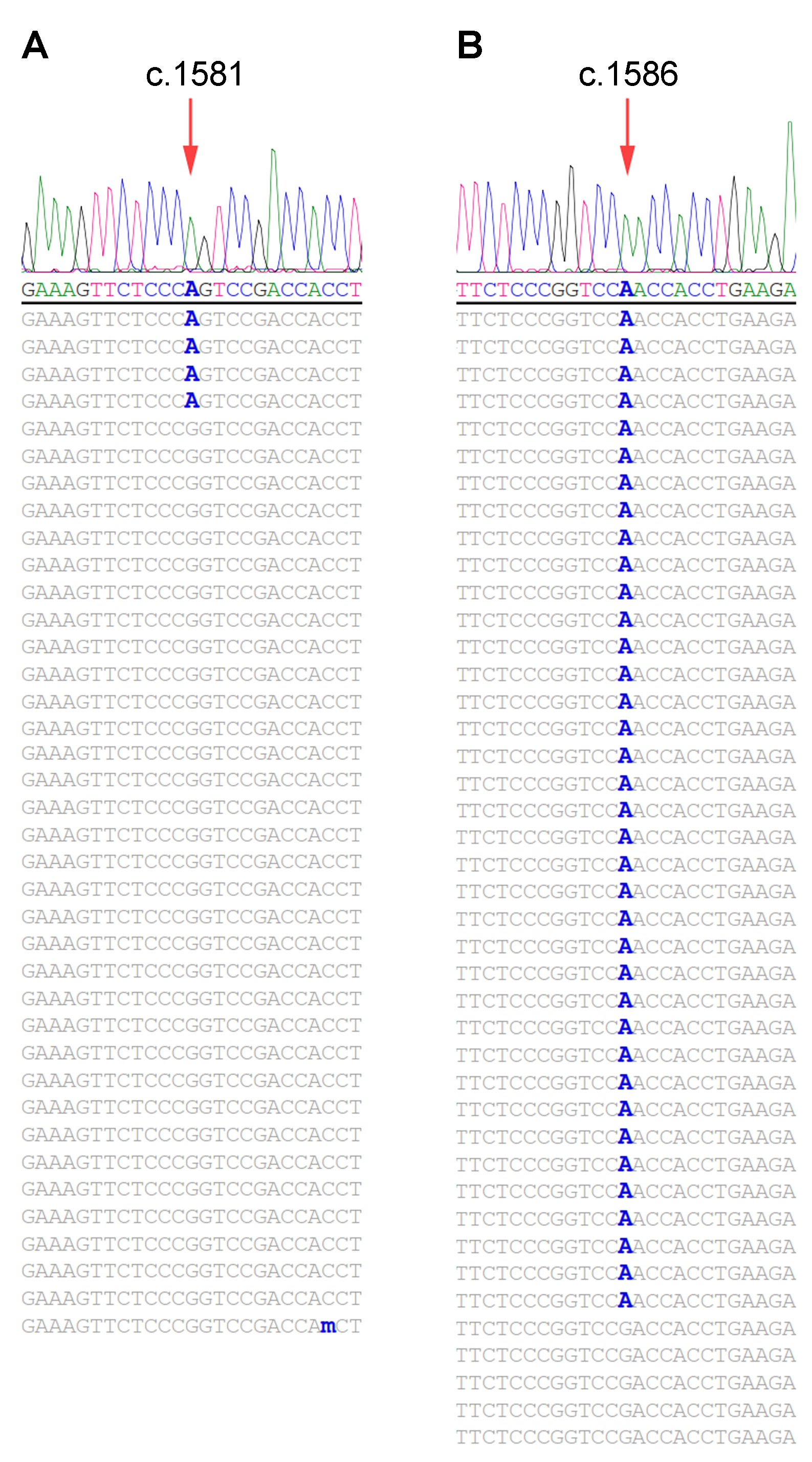

Supplement: S2 Fig — Knockdown experiments of the candidate genes were associated with some G-to-A alterations at non-conserved sites which were not observed in the control experiments, including changes at c.1581 (A) and c.1586 (B) associated with A3G-si and A3C-si, respectively. (TIF) [file pone.0120089.s002.tif]

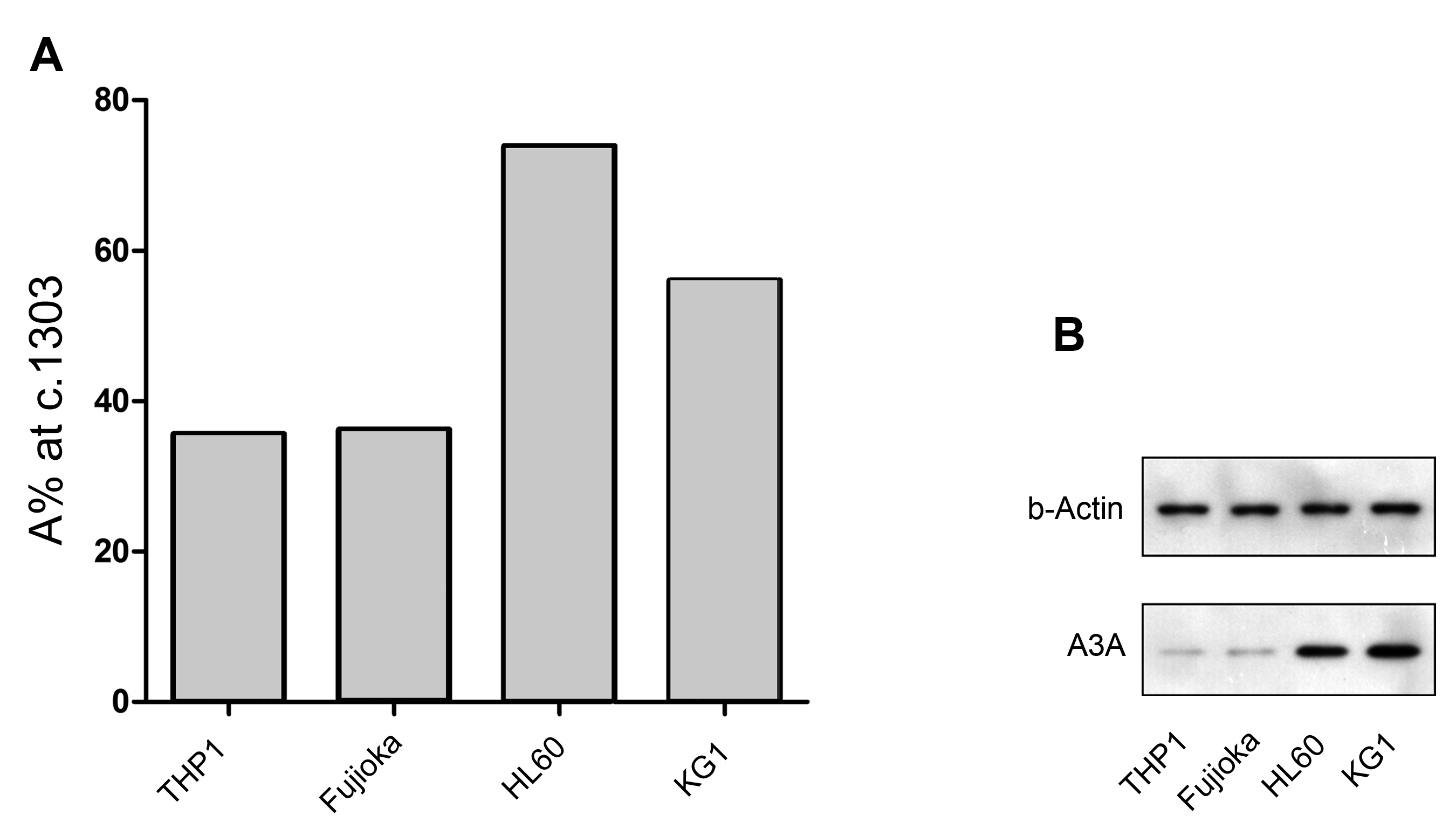

Supplement: S3 Fig — A. A% of at c.1303 in four myeloid cell lines as determined by clonal sequencing of the WT1 transcripts. B. Expression levels of A3A in corresponding myeloid cell lines compared to b-Actin as determined by Western blotting. (TIF) [file pone.0120089.s003.tif]

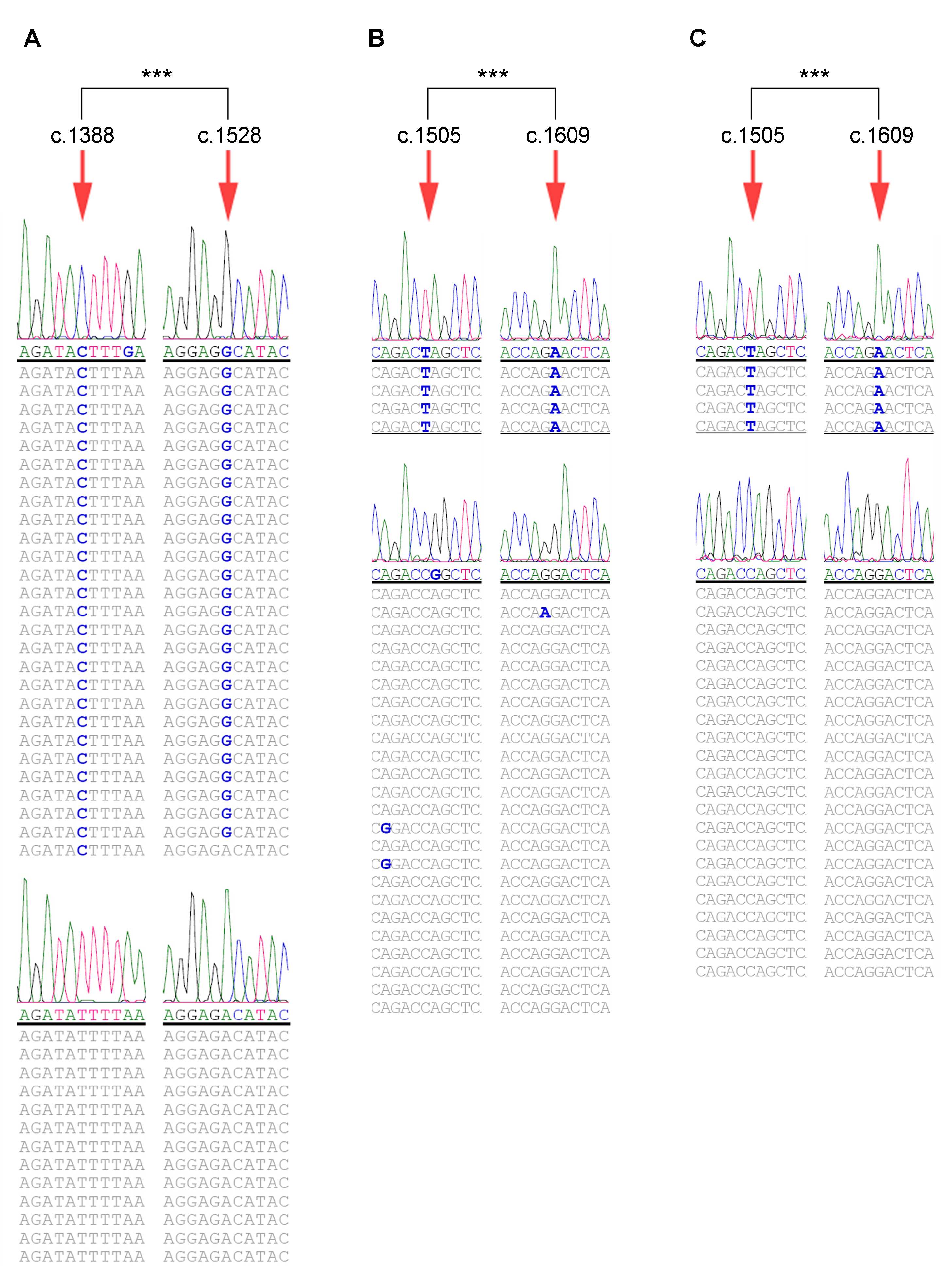

Supplement: S4 Fig — Sanger sequencing chromatograms of the WT1-cDNA clones are aligned and examined for the changes occurring in the same clones, i.e. in cis, with one altered chromatogram expanded for each of the associated events. Statistically significant associations are found for c.1388U>C and c.1528A>G (A), as well as c.1505C>U and c.1609G>A (B and C). *** P < 0.001. (TIF) [file pone.0120089.s004.tif]

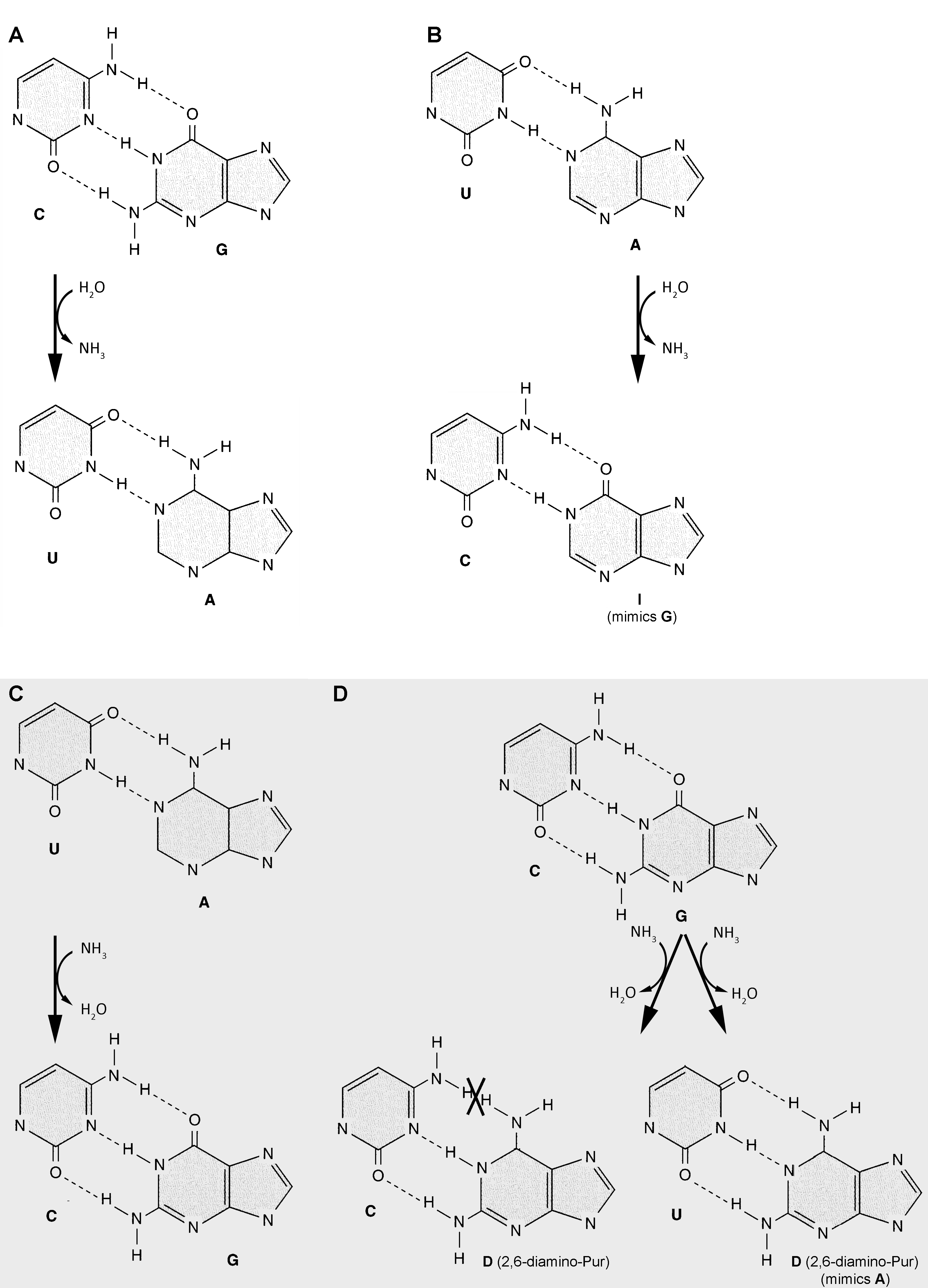

Supplement: S5 Fig — Classic mRNA editing is explained by deamination reactions converting C to U (A) and A to I, which mimics G (B), while proposed alternative mRNA editing model involves amination reactions converting U to C (C) and G to D, mimicking A (D). Each edited base is shown with its paired base. (TIF) [file pone.0120089.s005.tif]
