# Supplementary material for: APOBEC3A Is Implicated in a Novel Class of G-to-A mRNA Editing in WT1 Transcripts
Source: PLoS One. 2015 Mar 25;10(3):e0120089. doi: 10.1371/journal.pone.0120089 (PMC4373805; doi:10.1371/journal.pone.0120089)
Supplement: S1 Table — Gross G-to-A changes were seen in seven informative samples at c.1303 (underlined), including three homozygous GG samples (cb5, cb17, and cb21) with prominent A at c.1303, not explained by imbalanced allele expression. Sixteen samples showed prominent A at c.1586 (underlined). Overall, 17 samples showed G-to-A change at c.1303 and/or c.1586. * Samples with additional T-to-C alterations at c.1388 and c.1402. (PDF) [file pone.0120089.s006.pdf]

Position	g.39137 (rs16754)	c.1303	g.43522	c.1586
**cb4**	A	A	G	G
**cb10**	A	A	G	G
**cb12**	A	A	G	G
**cb14**	A	A	G	G
**cb3**	A	A	G	AG
**cb13**	A	A	G	AG
**cb6**	A	A	G	AG
**cb7**	A	A	G	AG
**cb18**	A	A	G	A
**cb2**	AG	AG	G	AG
**cb9***	AG	AG	G	AG
**cb19***	AG	AG	G	AG
**cb20**	AG	AG	G	AG
**cb11**	AG	AG	G	AG
**cb8**	AG	A	G	AG
**cb1**	AG	A	G	A
**cb15**	AG	A	G	A
**cb16**	AG	A	G	A
**cb21**	G	AG	G	G
**cb5**	G	A	G	AG
**cb17**	G	A	G	A
