# Supplementary material for: APOBEC3A Is Implicated in a Novel Class of G-to-A mRNA Editing in WT1 Transcripts
Source: PLoS One. 2015 Mar 25;10(3):e0120089. doi: 10.1371/journal.pone.0120089 (PMC4373805; doi:10.1371/journal.pone.0120089)
Supplement: S2 Table — (PDF) [file pone.0120089.s007.pdf]

Gene/ Transcript	siRNA	Sense Strand Sequence	Accession No.	Start Pos.	GC %	Score	Low Seed Freq?	Min # Mis-matches (α-sense)	Min # Mis-matches (sense)
ADAR	Adar1-si	GAAUAUGCCCAGUUCGCUAdTdT	NM_015841	1770	47	80	Yes	3	>3
ADARB1	Adb1-si	AGUCCAAGCUGGCGGCAAAdTdT	NM_001112	2414	58	90	Yes	3	2
ADARB2	Adb2-si	AGGUGAAGGAGAACCGCAAdTdT	NM_018702	577	53	78	Yes	2	3
AICDA	Aid-si	UGACUUACGAGACGCAUUUdTdT	NM_020661	640	42	72	Yes	2	3
APOBEC1	A1-si	CGGAAGAUCUGGCGAAGCUdTdT	NM_001644	176	58	79	Yes	3	2
APOBEC2	A2-si	GGCUGUAAACUGCGCAUCAdTdT	NM_006789	664	53	71	Yes	3	3
APOBEC3A	A3A-si1	GCAGUAUGCUCCCGAUCAAdTdT	NM_145699	896	53	81	Yes	>3	3
APOBEC3A	A3A-si2	GCUACGAAGUGGAGCGCCUdTdT	NM_145699	271	63	79	Yes	2	3
APOBEC3A	A3A-si3	GCAAGUCGCAAGAGCGGGAdTdT	NM_145699	118	63	74	Yes	3	2
APOBEC3B	A3B-si	GCUAUGAGGUGGAGCGCCUdTdT	NM_004900	704	63	81	Yes	2	3
APOBEC3C	A3C-si	AGAUCAGAAACCCGAUGAAdTdT	NM_014508	114	42	70	Yes	3	3
APOBEC3D	A3D-si	CCAAACGUCAGUCGAAUCAdTdT	NM_152426	589	47	72	Yes	3	3
APOBEC3F	A3F-si	GAAACACAGUGGAGCGAAUdTdT	NM_145298	310	47	89	Yes	2	2
APOBEC3G	A3G-si	GUUAUGAGGUGGAGCGCAUdTdT	NM_021822	1019	53	87	Yes	3	3
APOBEC3H	A3H-si	CAUCAAGGCUCACGACCAUdTdT	NM_001166003	412	53	81	Yes	3	3
APOBEC4	A2-si	CUGACAGGCACAACGCAUAdTdT	NM_203454	934	53	76	Yes	>3	3
Control	Cont-si	GCAAAGAAUCGGGCUAGUAdTdT	-						
Cont-Alx488	Alx-si	GCAUUCAGCUCGUAUGUAAdTdT	-						
