# Supplementary material for: APOBEC3A Is Implicated in a Novel Class of G-to-A mRNA Editing in WT1 Transcripts
Source: PLoS One. 2015 Mar 25;10(3):e0120089. doi: 10.1371/journal.pone.0120089 (PMC4373805; doi:10.1371/journal.pone.0120089)
Supplement: S3 Table — (PDF) [file pone.0120089.s008.pdf]

Set	Primer	Sequence	Gene	Amplicon
	Ws-Ex6f	CACGCACGGTGTCTTCAGAG	*WT1*	cDNA
1	Ws-Ex10r	GACAACTTGGCCACCGACAG	*WT1*	cDNA
	Ws-X7f5	CACTCTCCCTCAAGACCTACGTG	*WT1*	DNA
2	Ws-X7r5	GGTCCTTAGCAGTGTGAGAGCC	*WT1*	DNA
	Ws-X9f3	CTGGGGGACTGGGGAAATC	*WT1*	DNA
3	Ws-X9r2	GCCACGCACTATTCCTTCTCTC	*WT1*	DNA
